# Physicians’ attitudes and acceptance towards artificial intelligence in medical care: a qualitative study in Germany

**DOI:** 10.3389/fdgth.2025.1616827

**Published:** 2025-07-14

**Authors:** Sarah Negash, Jana Gundlack, Charlotte Buch, Timo Apfelbacher, Jan Schildmann, Thomas Frese, Jan Christoph, Rafael Mikolajczyk

**Affiliations:** ^1^ Institute for Medical Epidemiology, Biometrics and Informatics, Interdisciplinary Center for Health Sciences, Medical Faculty of the Martin Luther University Halle-Wittenberg, Halle (Saale), Germany; ^2^ Institute of General Practice & Family Medicine, Interdisciplinary Center of Health Sciences, Medical Faculty of the Martin Luther University Halle-Wittenberg, Halle (Saale), Germany; ^3^ Institute for History and Ethics of Medicine, Interdisciplinary Center for Health Sciences, Medical Faculty of the Martin Luther University Halle-Wittenberg, Halle (Saale), Germany; ^4^ Friedrich-Alexander-Universität Erlangen-Nürnberg, Institute for Medical Informatics, Biometrics and Epidemiology, Medical Informatics, Erlangen, Germany; ^5^ Junior Research Group (Bio-) Medical Data Science, Medical Faculty of the Martin Luther University Halle-Wittenberg, Halle (Saale), Germany

**Keywords:** artificial intelligence, physicians, attitudes, acceptance, medical care, healthcare

## Abstract

**Background:**

The role of artificial intelligence (AI) in medicine is rapidly expanding, with the potential to transform physicians’ working practices across various areas of medical care. As part of the PEAK project (Perspectives on the Use and Acceptance of Artificial Intelligence in Medical Care) this study aimed to investigate physicians’ attitudes towards and acceptance of AI in medical care.

**Methods:**

Between June 2022 and January 2023 eight semi-structured focus groups (FGs) were conducted with general practitioners (GPs) recruited from practices in the region of Halle/Leipzig, Germany, via email and postal mail, as well as with university hospital physicians from Halle and Erlangen, recruited via email. To conduct the FGs, a topic guide and a video stimulus were developed, including a definition of AI and three potential applications in medical care. Transcribed FGs and field notes were analyzed using qualitative content analysis.

**Results:**

39 physicians participated in eight FGs, including 15 GPs [80% male, mean age 44 years, standard deviation (SD) 10.4] and 24 hospital physicians (67% male, mean age 42 years, SD 8.6) from specialties including anesthesiology, neurosurgery, and occupational medicine. Physicians’ statements were categorized into four themes: acceptance, physician–patient relationship, AI development and implementation, and application areas. Each theme was illustrated with selected participant quotations to highlight key aspects. Key factors promoting AI acceptance included human oversight, reliance on scientific evidence and non-profit funding. Concerns about AI's impact on the physician-patient relationship focused on reduced patient interaction time, with participants emphasizing the importance of maintaining a human connection. Key prerequisites for AI implementation included legal standards, like clarifying responsibilities and robust data protection measures. Most physicians were skeptical about the use of AI in tasks requiring empathy and human attention, like psychotherapy and caregiving. Potential areas of application included early diagnosis, screening, and repetitive, data-intensive processes.

**Conclusion:**

Most participants expressed openness to the use of AI in medicine, provided that human oversight is ensured, data protection measures are implemented, and regulatory barriers are addressed. Physicians emphasized interpersonal relationships as irreplaceable by AI. Understanding physicians’ perspectives is essential for developing effective and practical AI applications for medical care settings.

## Introduction

As healthcare demands increase and workforce shortages emerge (1–3), the need for digital transformation to enhance efficiency and strengthen system capacity has become increasingly evident (4). In this context, artificial intelligence (AI) has the potential to optimize workflows and care processes, addressing key healthcare challenges (2).

The integration of AI into medical care is accelerating, offering solutions to enhance diagnostic accuracy, treatment efficiency, and personalized patient care (5). AI, defined as the use of machines to simulate human reasoning and problem-solving, is designed to tackle complex problems traditionally addressed by human experts (6). Subfields such as machine learning and natural language processing enable data analysis and task automation in healthcare, helping to reduce physicians’ workload and minimize errors from overwork (7–10). At the same time, the introduction of AI in healthcare presents challenges, including data privacy risks and reliance on potentially biased algorithms, which, when trained on non-representative data, can lead to inaccurate diagnoses (11, 12).

Across various medical specialties, a growing number of AI applications are emerging (11, 13). In dermatology, for instance, AI may support the diagnosis of skin lesions and could facilitate more efficient referrals from primary to secondary care (14, 15). However, if algorithms are trained on non-representative data, they may fail to accurately diagnose conditions in individuals with darker skin tones or those with uncommon skin conditions, thereby exacerbating existing health disparities (16). Additionally, several studies have reported that AI is increasingly applied in surgical practice, utilizing robotic-assisted systems to enhance precision, improve diagnostic accuracy, and support postoperative monitoring (17, 18). A well-known example is the Da Vinci system, which employs machine learning and computer vision to facilitate minimally invasive procedures (17, 19, 20). However, concerns persist regarding high acquisition and maintenance costs, longer operative times, and limited evidence demonstrating a clear clinical benefit (19, 20). Despite its growing integration, AI adoption remains inconsistent across medical specialties due to persistent ethical, technological, regulatory, liability, and patient safety concerns (9, 21). To address these challenges in the development and implementation of AI systems, appropriate regulatory oversight is indispensable (22). At the European level, the AI Act serves as a foundational legal framework, aiming to harmonize standards for safe, transparent, and human-centered AI across member states (23). Although regulatory frameworks such as the European AI Act aim to promote trustworthy AI, the actual readiness and confidence of physicians to use these technologies vary across countries. Similarly, prior studies from Saudi Arabia and South Korea have demonstrated variability in physician preparedness and confidence regarding the use of AI in clinical practice (24, 25).

Addressing these barriers requires the active involvement of physicians, whose acceptance is essential to achieving widespread adoption of AI-related technologies (26). Although physicians generally have favorable attitudes towards AI in medicine, several studies suggest that their experience and overall knowledge of AI applications remain limited (27–29). A recent systematic review reported that physicians and medical students were receptive to clinical AI, albeit with some concerns (27).

Despite these insights, research gaps remain in understanding physicians’ attitudes towards AI across different fields of medicine. In fact, the literature to date has primarily focused on individual medical fields (30, 31), thus failing to provide a comprehensive view of the medical profession as a whole. Additionally, while existing studies have predominantly employed quantitative methods (24, 29, 32), a qualitative approach may be more suitable for capturing physicians’ perspectives, as it allows for a more nuanced and exploratory investigation of their views (33). In this regard, conducting focus groups (FGs) can provide a deeper understanding of physicians’ views (34). As part of the PEAK project (Perspectives on the Use and Acceptance of Artificial Intelligence in Medical Care) (35), this study therefore aimed to explore physicians’ attitudes towards and acceptance of AI in medical care using a qualitative approach.

## Methods

### Participants and data collection

This qualitative study is part of the PEAK project, an explorative sequential mixed-methods study designed to explore physicians’ and patients’ attitudes towards AI in medical care. For this part, we included general practitioners (GPs) and hospital physicians to represent two distinct levels of care within White et al.'s healthcare pyramid: primary care and tertiary care (36, 37). By selecting these two groups, which differ fundamentally in their clinical routines, patient populations, and potentially also in their perspectives (38), we aimed to capture a broad spectrum of perspectives and attitudes towards AI in medical care (38, 39). Between June 2022 and January 2023, a total of eight semi-structured FGs were conducted with GPs and university hospital physicians. GPs were recruited from the research network RaPHaeL (Research Practices Halle-Leipzig) (40), and the Medical Association of Saxony-Anhalt, Germany, via email and postal mail. Similarly, hospital physicians were recruited from the University Hospitals Halle (Saale) and Erlangen, Germany, via email. FGs consisted of three to six participants each and were conducted separately for GPs and hospital physicians. Prior to the FGs, the participants’ socio-demographic data, level of further education, duration of medical practice, specialization, experience in their specialization, and technology affinity were collected. The latter was assessed using three items on Perceived Technology Competence from a standardized questionnaire (41). Information about the study was communicated both orally and in writing to participants before the FG. They were informed about the voluntary nature of their participation, the lack of financial compensation, and their right to refrain from answering questions or to terminate the focus group without providing a reason. No monetary incentives were offered for participation; however, participants were informed in advance that light refreshments would be provided during the focus groups. Study participants gave their written informed consent. After introducing a stimulus through a video, the moderator used questions from the FG guide to prompt the discussion, ensuring that all participants were actively engaged in the discussion. The FGs were moderated by two researchers, both of whom were largely unfamiliar to the participants. Prior to the FGs, participants were informed about the moderators’ backgrounds and the aim of the study. They were given space to express their opinions openly and without disruptions. All FGs were audio-recorded, with duration ranging between 88 and 135 min, and notes were taken by additional researchers. FGs were conducted until thematic saturation was reached, which was assessed through an emergent, iterative process. Saturation was considered achieved when no new relevant themes, insights or codes emerged from the data. All data were handled confidentially.

### Outline of topic guide and application examples

A topic guide was developed using literature from a PubMed search and guided by Helfferich's methods (42) and Krueger and Casey's approach to focus group guides (43). It included open-ended questions designed to explore attitudes towards AI in medical care, including factors promoting and hindering physicians’ acceptance of AI, effects on the physician–patient relationship, challenges in implementing AI, and areas of application for AI. To stimulate the FGs, a video presentation was shown that defined AI and highlighted three potential healthcare applications: (a) diagnosis: symptom check via Ada app (44); (b) treatment: alternative medication plan (45); (c) process optimization: voice documentation (46). The example sequence was varied across groups to reduce bias, with all materials pretested.

### Data analysis

The audio-recorded FGs were transcribed verbatim. To ensure anonymity, any information that could identify participants was removed from the transcripts, and pseudonyms were used to replace real names, ensuring that identification was no longer possible. The textual material was analyzed using a content analysis approach linked to Mayring (47) by systematically assigning physicians’ attitudes to a codebook. It was developed collaboratively by four researchers independently coding a representative FG and discussing and refining the codes until consensus was reached. The codebook was applied to all FGs by three of the four researchers and modifications were discussed after each FG until consensus was achieved; new codes were applied to all FGs. Main themes were generated deductively based on the FG guide, while subthemes were identified inductively from the data. All researchers used MAXQDA software. To enhance methodological credibility, the coding was conducted iteratively with regular team discussions to resolve discrepancies and refine the codebook. Consensus was achieved through collaborative coding and thorough review. Member checking was not performed, as the focus of the analysis was on thematic patterns across groups rather than on individual perspectives.

## Results

### Participant characteristics

39 physicians participated in eight FGs, including 15 GPs (80% male, mean age 44 years, SD 10.4) and 24 hospital physicians from various specialties, such as anesthesiology, neurosurgery, and occupational medicine (67% male, mean age 42 years, SD 8.6). The majority of participants (60.5%) had a high affinity for new technology and around one third of participants (34.9%) a medium affinity. A more detailed description of the sample is given in Table 1.

**Table 1:** Participants’ characteristics (*n* = 39).

Characteristic	Frequency (%) or mean (SD)
Age, mean (SD)	43 (9.2)
Gender	
Female, *n* (%)	11 (28.2)
Male, *n* (%)	28 (71.8)
Medical role and career stage	
Doctor in further training, *n* (%)	10 (25.6)
Medical specialist, *n* (%)	16 (41)
Senior physician, *n* (%)	12 (30.8)
Chief physician, *n* (%)	1 (2.6)
Specialization	
General medicine, *n* (%)	15 (38.3)
Occupational medicine, *n* (%)	3 (7.7)
Radiology and radiotherapy, *n* (%)	2 (5.1)
Hygiene and environmental medicine, *n* (%)	2 (5.1)
Cardiology, *n* (%)	1 (2.6)
Hematooncology, *n* (%)	1 (2.6)
Nephrology, *n* (%)	1 (2.6)
Anesthesiology (and intensive care medicine with emergency medicine), *n* (%)	3 (7.7)
Surgery, *n* (%)	1 (2.6)
Visceral surgery, *n* (%)	1 (2.6)
Trauma surgery, *n* (%)	1 (2.6)
Neurosurgery, *n* (%)	3 (7.7)
Dentistry, *n* (%)	1 (2.6)
Neurology, *n* (%)	2 (5.1)
Child and adolescent psychiatry, *n* (%)	2 (5.1)
Duration of medical practice (in years)	
0–10, *n* (%)	15 (38.5)
11–20, *n* (%)	14 (35.9)
>20, *n* (%)	10 (25.6)
Years in specialization	
0–10, *n* (%)	11 (28.3)
11–20, *n* (%)	13 (33.3)
>20, *n* (%)	5 (12.8)
Not specified, *n* (%)	10 (25.6)
Affinity for new technology	
Low, *n* (%)	2 (4.6)
Medium, *n* (%)	15 (34.9)
High, *n* (%)	26 (60.5)

### Themes and subthemes

**Figure 1:**
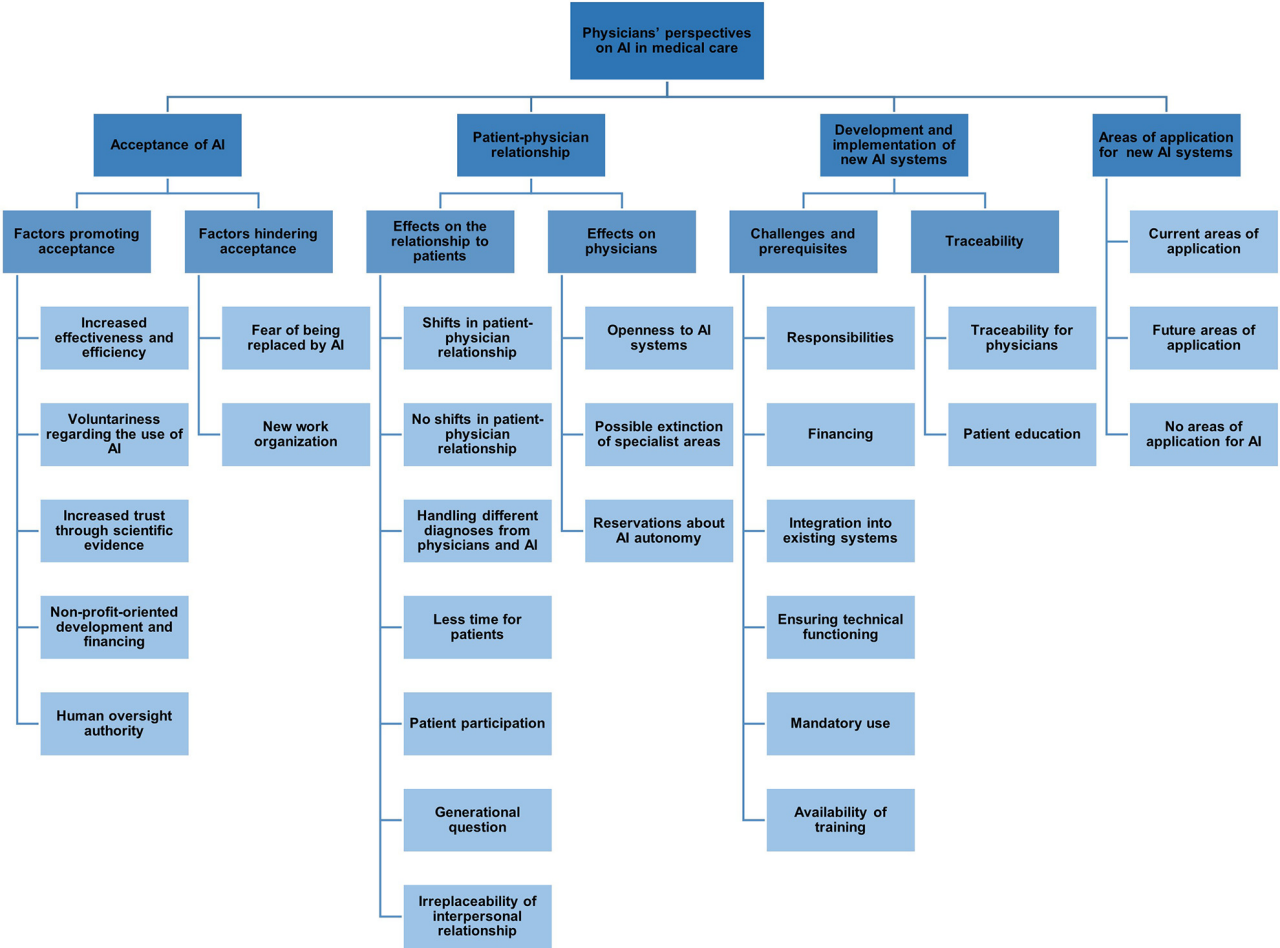
Thematic map of the codebook: physicians’ perspectives on artificial intelligence (AI) in medical care.

The results of this study are organized into four main themes: (1) acceptance, (2) physician–patient relationship, (3) development and implementation of new AI systems, and (4) areas of application for AI. An overview of the thematic structure is illustrated in Figure 1. Each theme and its subthemes are described in detail below; quotes can be seen in Tables 2–5.

**Table 2:** Subthemes, codes and illustrative quotes for the theme acceptance of AI.

Subthemes and codes	Illustrative quotes
Factors promoting physicians' acceptance of AI	
Increased effectiveness and efficiency for physicians	"If it goes well, it could potentially add value, and then we might also estimate the acceptance to be somewhat higher. … We collect data, the AI system processes it and gives us something. And if that aligns with what we've done so far, or maybe even performs better, why not?” (FG3-Ä.4)
Voluntariness regarding the use of AI	"It must be voluntary, what you said, and there must be a benefit, both a health-related and a financial one. That's how a system gets established.” (FG2-ALL.5)
Increased trust through scientific evidence	"I also believe that if it was perhaps confirmed by higher centers [..] as certified. If the WHO now says, yes, this is a system that we also work a lot with, where we also feed in our data, and where well-known organizations are behind it, then I would inherently have more trust in it. [..] I think that would simply help me. If I know that high-ranking scientists are involved and have worked on it.” (PRE.1FG-Ä3)
Non-profit-oriented development and financing	"I think I would rather take the approach of looking at what the intention behind the development of the system was and by whom. So, is there a corporation behind it that wants to maximize profit and make as much money as possible with this AI or something like that? Or is it something more driven by the common good?” (FG4-Ä.4)
Human oversight authority	"I can't fully understand the program. I'm not an IT specialist, I'm a medical doctor. But in any case, there needs to be a regulatory body, which was already mentioned earlier, that I can trust. And whether that's some sort of government entity or more of an open-source process, that would need to be discussed.” (FG4-Ä3) “The developer, as you just mentioned, is responsible for ensuring that it functions technically. This brings us back to quality management, reevaluation, process monitoring, and so on. These processes must always take place. It is self-learning, meaning that a new code and files are generated in the background. Someone needs to oversee this, and that falls to the technical side.” (FG2-ALL.5)
Factors hindering physicians’ acceptance of AI	
Fear of being replaced by AI	"I have repeatedly observed that there are huge fears surrounding this topic, especially because neurophysiologists, whose main job is to sit in front of a screen and monitor the situation, are actually afraid of being driven out of the operating room by evil machines. And some of them have completely vague ideas about how advanced these technologies actually are and how capable they truly are.” (FG3-Ä5)
New work organization	"I'll start with something really simple first, it has to be easily accessible. Because there are already various solutions and scientific approaches. And I don't use a lot of them because they simply take too much time. … It's often like this: if I had the option to do something better with a different software, but I would need to take my data and transfer it from one software to another. That alone could be enough for me to say, maybe not. It takes extra time again. That doesn't even have anything to do with AI…If it's based on documentation that's already there, then it will certainly work better. But then the question is how it's presented. Do I first have to scroll through dialogues? Or is it close to what is reflected in clinical practice?” (FG2-Ä.1)

Abbreviations: AI, artificial intelligence; FG, focus group; Ä, hospital physician; ALL, general practitioner. Identifiers are pseudonyms assigned to anonymized participants.

**Table 3:** Subthemes, codes and illustrative quotes for the theme patient-physician relationship.

Subthemes and codes	Illustrative quotes
Effects on the patient-physician relationship	
Shifts in the patient-physician relationship due to AI use	"These programs can essentially generate a report independently and then determine whether medical diagnostics should be initiated or not. And of course, the big question is, is this helpful? Is this the right path, or is the right path to improve medical care? That's certainly a major question. But this also leads away from the doctor-patient interaction. Maybe not here, but in general, speaking broadly, where AI might lead or could go.” (PRE.1FG-Ä6)
No shifts in the patient-physician relationship due to AI use	"And when the patient goes through the diagnostic process, they don't even realize at that moment that it's AI, but rather, it feels like a normal routine procedure to them.” (FG1-ALL.1) “But if this person—this person, in quotation marks, this artificial person—stood next to me or even between us, I wouldn't be too scared in the short term…I think if it has this benefit-oriented character, I would say that a patient being treated by a doctor who openly says: ‘I use AI but I'm still here for you, I make the diagnoses, I explain it to you,’ that would actually help me move forward. I wouldn't have any concerns about that.” (FG2-Ä3)
Dealing with different diagnoses from physicians and AI	"Well, if I weren't, if I weren't generally willing to accept that, then I wouldn't have to use AI at all. Because, as we've said, that's part of the concept. That there are findings that I wouldn't have come up with using my own logical conclusions. That means I can certainly question it, and maybe I can even verify it, but I have to live with the fact that it will sometimes be like that. And then accept it and trust it.” (FG3-Ä2)
Less time for patients	"We will focus more on technology and less on the patient. I don't believe it will lead to more time for the patient. (FG1-Ä.1: Exactly, not at all.)” (FG1-Ä3)
Patient participation	"Ideally, there might also be an interface for the patients. Something in the sense of Patient Reported Outcome Measures. In other words, where the patient can continuously integrate things into this entire, well, AI system.” (FG1-Ä3)
Generational question	"Yes, that's a generational problem. I mean, of course, it's the older people who, let's say, didn't grow up with computer technology. Naturally, they will continue to be critical of it. And the younger generation will grow up with it, and it will be so commonplace that we won't have any communication problems at all.” (FG4-Ä4)
Irreplaceability of interpersonal relationships with physicians	"I think what AI can't do is interact with patients. It can't replace the emotional and, so to speak, the non-verbal interaction with the patient. And it also can't grasp the socioeconomic and social-medical problems the patient is dealing with.” (FG1-ALL.4)
Effects on physicians	
Physicians open to AI systems	"I believe it would -at first glance- simplify things. Because you have something tangible right from the start, which you can work on relatively quickly with the patient. … So, you're skipping the first steps because the technology takes care of that. And then you move on to the second step. So, I would actually find that rather simplistic.” (FG3-ALL.5)
Extinction of specialist areas possible	"Every profession has repetitive, identical, recurring tasks, some more, some less. … But anything that repeats itself and doesn't require creativity can, in principle, be automated and taken over by AI.” (FG3-ALL.3) “You can also summarize certain individual processes that already exist. Dermatologists, for example, if they use AI to assess liver spots, you don't need a dermatologist for that. Or we had the example of radiology earlier. Bronchial carcinoma screening works with AI. Yes?” (FG1-ALL.1)
Reservations about AI autonomy	"Exactly. Data protection is one thing. But also, you're sharing all these very intimate details within a system, and you don't know where it's going. And I think that responsibility shouldn't be completely handed over to the machine. … But, I mean, there's still a lot of responsibility that is still left to us, which is the challenge, I'd say, for ourselves. To ask, how much of my own pride can I hand over to a machine? There needs to be a lot of rethinking. If that happens, then there's room for openness. But I don't think humans are wired to trust a machine with something like that. (laughs)” (FG3-ALL.4)

Abbreviations: AI, artificial intelligence; FG, focus group; Ä, hospital physician; ALL, general practitioner. Identifiers are pseudonyms assigned to anonymized participants.

**Table 4:** Subthemes, codes and illustrative quotes for the theme development and implementation of new AI systems.

Subthemes and codes	Illustrative quotes
Implementation challenges and prerequisites	
Different areas of responsibility in implementation	"I believe the responsibility lies with everyone collectively because everyone is involved in this process. Those who have to program it must ensure that the data is collected, processed, and interpreted appropriately. Those who use AI should hopefully only use it as a tool, as a supplement, but not as the sole diagnostic method. And the patient, who also interacts with the AI, must seriously provide information with a certain level of prioritization and severity, so that the AI can produce the best results. In the end, the responsibility rests with everyone. But the danger is, of course, that the responsibility will eventually be shifted onto the doctor, who is the final point of control.” (FG1-ALL.1)
Financing of AI systems	"And that one receives financial support for such matters, so that the burden doesn't fall entirely on the practices.” (FG2-ALL.5) “Those who ultimately pay the money, meaning the health insurance companies from the common pot, will always have such systems and will then calculate things differently. … That's a different level, a political level, and a systemic question: how to allocate the money, who pays for it, and how we can compensate it?” (FG2-ALL.5)
Integration into existing systems	"And that all systems are compatible with each other. There will certainly be different companies involved. And maybe at some point, you might switch providers because the next company offers a better deal or something like that, everything must be compatible with everything. That's very important. Also, the software used in hospitals and in private practices.” (FG4-Ä2)
Ensuring technical functioning	"I believe this is also a technical development, which brings us back to the point that it needs to be a system that is not tested in our practice but must work effectively before it is implemented on a large scale. It must be clear how the process functions.” (FG2-ALL.5)
Mandatory use	"It must be mandatory for everyone, including for hospitals. We are already receiving electronic medical reports, and artificial intelligence could be effectively utilized here to recognize when a diagnosis is mentioned, which could then be automatically integrated into the practice management software. Again, the question arises regarding data protection.” (FG2-ALL.5)
Availability of training	"I believe that hospitals and especially medical practices need more training on this topic. The large internet companies have security personnel for these matters, but medical practices do not prioritize this. They focus on keeping the system running and don't hire additional help for security. I think this creates a significant security risk, as data can be accessed relatively easily.” (PRE.1FG-Ä4)
Traceability in the implementation of new AI systems	
Traceability for physicians	"Transparency, as we mentioned earlier, is key. The traceability of AI's results holds great importance.” (FG4-Ä5) “Maybe somewhat gradated. I could imagine that there are areas where I can still keep up, where I think I might be better than the AI. But then with those very complex applications, where I can't follow anyway and can't even tell anymore if something is plausible or not, the question doesn't really arise anymore.” (FG3-Ä.2)
Patient Education	"I would have to tell the patient that we now have an additional AI tool that we are using as support. On the one hand, I think the patient has a right to know where the information is coming from or how it is being generated. They have a right to that. And on the other hand, we can also frame this positively by saying: ‘For various reasons, we are now using AI for your safety and to manage the workflow’.” (FG1-ALL.1) “If the systems are established and prove to be helpful, they will be accepted. That means they will simply be accepted, even by patients. They need the progress to provide therapy or treatment, so to speak. And I hope that the patient is interested in their recovery and not in the technology behind it.” (FG1-Ä.2)

Abbreviations: AI, artificial intelligence; FG, focus group; Ä, hospital physician; ALL, general practitioner. Identifiers are pseudonyms assigned to anonymized participants.

**Table 5:** Codes and illustrative quotes for the theme areas of application for AI.

Codes	Illustrative quotes
Current areas of application	"In the newer software versions for radiation planning, AI components are already integrated. These are learning systems that, based on previously processed cases, make a suggestion for automatically defining these contours [tumor target area and healthy tissues].” (FG3-Ä.2)
Future areas of application	"One major topic for us is certainly differential diagnostics and screening.” (FG2-Ä.1) “Well, for example, let's say a dementia screening—it's always quite time-consuming for us. Most of the time, we no longer have any mid-level medical staff, and we're all understaffed, really. So, if for instance, an older person came in and could already communicate with a computer or some kind of system, and we could find out whether they have trouble finding words or any other memory issues, I wouldn't find that bad.” (FG2-ALL.4)
No areas of application for AI	"Well, I think where I really see the limits is in the final decision-making. I don't think artificial intelligence will ever reach that point. At least, I hope it never gets to the point where a patient inputs all their data into an app, and then some robot spits out a pill. I really hope it never comes to that. Rather, I believe the ultimate course of action, the final treatment, should come from a doctor, or from nurses, or whoever is responsible. But in that last step, I think that's where the limits of artificial intelligence are reached.” (PRE.1FG-Ä3) “I see the absolute limit of AI in psychotherapy, in talk therapy. I see no way that artificial intelligence could do that. Or maybe I just lack the imagination to see if that might be technically possible someday. But I don't think so. I believe that talk therapy is an absolute boundary. Everything that is psychotherapy.” (FG3-ALL.6)

Abbreviations: AI, artificial intelligence; FG, focus group; Ä, hospital physician; ALL, general practitioner. Identifiers are pseudonyms assigned to anonymized participants.

### Theme 1: acceptance

#### Subtheme 1: factors promoting physicians᾽ acceptance of AI

In the FGs, participants considered a substantial increase in effectiveness and efficiency in their medical work as a crucial precondition for accepting the use of AI in their daily routine (Table 2). Examples mentioned in this context were improvements in diagnostic accuracy and in personalized treatment. Physicians emphasized the importance of adopting AI tools at their own pace and discretion to maintain autonomy in their decision-making. Furthermore, participants noted that trust in AI and willingness to adopt it in practice increase when systems are supported by strong scientific evidence, reinforcing confidence in the efficacy and safety of these technologies. Some participants also highlighted the need for AI systems to be developed and financed without profit motives, expressing concerns that such motives could compromise the integrity of AI applications. Physicians advocated for models that prioritize ethical considerations and patient welfare over financial gain. Additionally, most participants emphasized the importance of ensuring that AI systems in the medical field do not operate autonomously, but are instead supervised and controlled by experts and regulatory bodies, highlighting the need for human oversight to ensure safety, accuracy, and ethical standards in medical applications.

#### Subtheme 2: factors hindering physicians’ acceptance of AI

Participants expressed concerns about the potential for AI to replace aspects of their professional roles. Some suggested that AI could eventually perform tasks traditionally carried out by physicians, leading to fears of job displacement and a reduction in the value of their expertise. Framing AI as a replacement for physicians could lead to resistance from both medical professionals and patients. Instead, they suggested that framing and designing AI as a supportive tool for physicians would be more likely to gain acceptance and be successfully integrated into medical practice. Some participants, particularly GPs, perceived the new organization of work and the additional workload that might result from the introduction of AI systems as a barrier to their acceptance.

### Theme 2: physician–patient relationship

#### Subtheme 1: effects on the physician–patient relationship

The potential shift in the physician–patient relationship as a result of the integration of AI systems was another concern raised by some participants (Table 3). They expressed that AI might alter this relationship, particularly in terms of trust and communication, emphasizing the importance of maintaining interpersonal relationships with their patients. Another recurring theme in this context was the fear that the use of AI could result in less time for patients, as the focus may shift toward technology-driven diagnostics and treatment planning.

In contrast, other physicians indicated that the integration of AI would not lead to significant changes in the physician–patient relationship. They regarded AI as a supportive tool that would enhance decision-making without compromising key elements such as trust, communication, and empathy, which they believed remain central to physicians. While acknowledging the growing role of AI in healthcare, participants emphasized that the human connection with patients, which they identified as a key element of patient-centered care, cannot be replaced by machines. Some physicians reflected on the challenges of dealing with different diagnoses from physicians and AI, highlighting potential confusion or uncertainty for patients. Discussions also revealed that the effects of AI on the physician–patient relationship might vary across generations, with younger patients perhaps being more comfortable with AI, while older generations might place more value on personal interaction with their physicians.

#### Subtheme 2: effects on physicians

Regarding the effects of AI on physicians themselves, the majority of participants expressed openness towards incorporating AI systems into their practice, recognizing the potential benefits for diagnosis and treatment. However, some physicians voiced reservations, particularly regarding the autonomy of AI systems, raising concerns about the extent to which AI should be allowed to make independent decisions in patient care.

### Theme 3: development and implementation of new AI systems

#### Subtheme 1: implementation challenges and prerequisites

Physicians discussed the required prerequisites and various challenges related to the development and successful implementation of new AI systems in medical practice (Table 4). A central topic was the clarification of their own and other stakeholders’ responsibilities. Participants expressed their responsibility for final decision-making, while they considered AI developers responsible for ensuring the technical robustness of AI systems and for training their systems with diverse datasets to prevent discrimination in medical applications. Opinions diverged, however, regarding the level of responsibility attributed to patients. On the one hand, physicians suggested that patients were often unaware of the tools and technologies physicians used in the background, and that such understanding could not reasonably be expected. On the other hand, they argued that patients should be regarded as active participants, whose consent and understanding of AI's role in their care were essential.

Physicians highlighted the challenge of ensuring collective responsibility in the implementation of AI, emphasizing the crucial roles of developers, physicians, and patients, while noting the risk that ultimate responsibility could be disproportionately shifted onto physicians as the final point of control. Another challenge mentioned by participants, particularly GPs, was the financing of AI systems. Several GPs noted that they might need to cover the costs of these systems for their practices, which could pose a financial burden. Both GPs and hospital physicians emphasized the importance of seamless compatibility with current medical workflows and the need for appropriate technical functioning of AI systems. Additionally, most participants advocated for the mandatory use of AI systems across all medical facilities (including practices and clinics) to promote standardization in medical care, provided that robust data protection measures are in place. Physicians also highlighted the necessity of adequate training to ensure the safe integration of AI systems into their daily work and raised concerns about potential security risks for medical practices associated with AI implementation.

#### Subtheme 2: traceability in the implementation of new AI systems

While some physicians viewed full traceability as difficult to achieve due to the sophisticated nature of AI systems, others emphasized that comprehensibility and transparency were essential to ensure accountability. Opinions were similarly divided regarding patient education, ranging from the importance of informing patients about the use and benefits of AI systems in their treatment to facilitate informed decision-making, to no need to explain the technology and its underlying principles once AI systems are integrated into routine practice.

### Theme 4: areas of application for AI

Finally, physicians discussed their perspectives on the current and potential future applications of AI in healthcare (Table 5). They indicated that AI is currently being introduced or has already been applied in several areas, including radiology and dermatology. In particular, several physicians highlighted the use of AI in radiotherapy, emphasizing its role in enhancing workflow efficiency by automating tasks such as the contouring of organs at risk and tumors in imaging scans. In dermatology, a few others noted AI's role in the early detection of skin cancer. Participants considered these application areas particularly well suited for AI integration due to the high degree of standardization and the availability of image-based data in these specialties.

Furthermore, physicians identified several potential future application areas for AI in medical care. They particularly highlighted the areas of early diagnosis and screening as promising fields for AI integration. According to the participants, AI systems could be valuable in detecting diseases at earlier stages, which would improve patient outcomes and reduce the burden on healthcare systems. In the field of neurology, they frequently noted AI's potential role in the early detection of diseases such as Alzheimer's, emphasizing that its predictive capabilities could meaningfully advance preventive strategies across various medical specialties. Physicians suggested that AI could help to tailor treatments to individual patients by analyzing large datasets to predict treatment outcomes more accurately. Additionally, AI's potential to assist in surgical planning and real-time decision-making during procedures was highlighted as an area that could enhance precision and patient safety.

In contrast, participants expressed clear hesitations about certain areas where they do not see AI playing a major role in healthcare. Mental health, particularly psychotherapy, was one domain where they felt AI would be less effective, citing the importance of human empathy and the nuanced understanding required in therapeutic relationships. Similarly, they considered AI unsuitable for use in direct patient care, where the personal touch and human connection are crucial for providing comfort and emotional support to patients. Another area where physicians were skeptical about AI's potential was in ultimate decision-making, especially in complex medical cases where ethical considerations and the expertise of experienced physicians are paramount. Participants strongly emphasized that human interaction is irreplaceable in many aspects of healthcare, stressing that the physician–patient relationship, built on trust, communication, and compassion, cannot be replicated by machines. In discussing application areas, physicians also addressed why AI remains in its early stages in many fields. The reasons mentioned included insufficient data sources for training AI systems, data protection concerns, and technical challenges.

## Discussion

This study explored physicians’ attitudes towards and acceptance of AI in medical care, drawing on perspectives from multiple medical specialties. While most themes were discussed similarly across GPs and hospital physicians, certain differences became apparent. For instance, GPs, operating at the primary care level, voiced greater concern about the practical and financial aspects of AI implementation, which tend to be less pressing for hospital physicians working in tertiary care settings, where implementation is often handled at the institutional level. Overall, physicians’ acceptance of AI was influenced by perceived benefits such as increased efficiency and diagnostic accuracy, but also shaped by concerns regarding AI autonomy, changes to the physician–patient relationship, and broader ethical implications. Participants emphasized the need for human oversight, scientific validation, and the establishment of ethical and regulatory safeguards. While there was skepticism about using AI in empathy-driven domains such as psychotherapy and caregiving, participants recognized potential applications in early diagnosis, screening, and data-intensive, repetitive processes.

In terms of factors promoting acceptance, physicians highlighted the potential of AI to substantially enhance effectiveness and efficiency in their medical work, which aligns with previous findings emphasizing AI's role in improving diagnostic accuracy and personalized care (5, 48). Participants highlighted the need for physician-driven AI adoption to safeguard professional autonomy, aligning with broader concerns in the literature on maintaining professional independence while integrating new technologies (49). Furthermore, trust in AI was closely linked to scientific validation, with physicians advocating for robust evidence to support the efficacy and safety of these systems. Additionally, participants strongly favored human oversight of AI systems, reflecting broader discussions about the indispensable role of expert regulation in mitigating potential risks and maintaining high ethical standards (12, 50). On the other hand, barriers to acceptance were closely linked to fears of being replaced in certain tasks and the potential devaluation of physicians’ expertise. These findings align with prior research indicating that framing AI as a supportive tool rather than a replacement is key to fostering acceptance among healthcare professionals (51). Notably, GPs expressed concerns about potential disruptions to their workflow, highlighting the need for seamless integration of AI systems into existing practices. Similarly, Shamszare et al. (52) suggested that optimal integration strategies could foster clinicians’ trust, mitigate perceived risks and workload, and enhance acceptance of AI-assisted clinical decision-making. This underlines the importance of addressing organizational challenges to support the adoption of AI in medical care.

Our results revealed participants’ concerns that AI could significantly influence the physician–patient relationship by compromising trust and communication, underscoring the importance of preserving the interpersonal connection central to patient-centered care. These concerns align with findings from Kerasidou (53) and Inanaga et al. (54) who highlighted the critical role of empathy and trust in patient satisfaction and treatment adherence. While AI can streamline clinical processes (55), some participants worried this could detract from direct patient interaction. Participants also highlighted the issue of responsibility in medical decision-making, emphasizing the need to clarify the responsibilities of all stakeholders while ensuring that physicians, alongside patients, remain the ultimate decision-makers. This perspective reflects a broader concern within the medical community about the ethical and legal implications of AI integration and was identified by physicians as a prerequisite for the successful implementation of AI systems in medical practice. Clear regulations delineating responsibilities are essential to ensure legal protection and maintain trust in medical decision-making (56, 57). This perspective aligns with the concerns raised by the German Medical Association (“Bundesärztekammer”) in a recent statement, which underscores the importance of addressing both technical and ethical challenges in the implementation of AI across various medical environments (58). These include the need for robust validation of AI models across diverse patient populations, transparency to ensure comprehensibility for physicians, and a regulatory framework that enables innovation while safeguarding physicians’ responsibility for diagnosis and treatment. Similar to previous findings in the literature (21, 59), our results indicated that while physicians, developers, and patients were all identified as key stakeholders in AI implementation, the precise allocation of responsibilities and liabilities remains unresolved. Physicians were predominantly seen as ultimately responsible for clinical decisions, while AI developers were expected to ensure technical robustness and minimize biases by utilizing diverse datasets. Perspectives on patients’ roles varied; some saw them as largely unaware of the technologies in their care, while others stressed the need for active participation and understanding of AI's role in treatment. Consistent with this, a qualitative study on patient perspectives regarding engagement in AI highlighted the significance of meaningful patient involvement, suggesting that it should serve as the gold standard for AI application development (60). These findings reflect existing concerns about regulatory gaps, given that current liability frameworks fail to clearly define stakeholder responsibilities in AI-driven healthcare, calling for urgent regulatory updates (61).

Echoing findings from a previous study (62), GPs particularly highlighted concerns about the financing of AI systems, including acquisition and operational costs, as well as the need for financial support for implementation. Beyond these concerns, the literature highlights potential benefits of implementing AI in medical practice, such as cost reductions through increased efficiency, automation of manual tasks, and lower expenses related to misdiagnoses or late diagnoses (63, 64). Furthermore, explainability and traceability of AI systems were recurring themes. In this context, many physicians emphasized the importance of comprehensible AI processes for building trust, while acknowledging the challenges posed by the ‘black box’ nature of AI. This is consistent with findings from the study by Sangers et al. (50), which focused on the views of GPs and dermatologists and found that they struggled to understand the rationale behind algorithmic decisions, making it difficult to assess their accuracy.

When participants discussed current and potential future applications of AI in healthcare, they emphasized its diverse capabilities, particularly in data-intensive specialties like radiology and dermatology, with a focus on workflow efficiency enhancement. These findings align with earlier studies emphasizing AI's compatibility with standardized, image-based fields (15, 30, 65). Mental health, on the other hand, particularly psychotherapy, was seen as reliant on human empathy and nuanced understanding, making AI less suitable. Similarly, caregiving roles requiring emotional support were viewed as unsuitable for AI implementation. While these concerns align with previous studies emphasizing the importance of human interaction in these domains (66, 67), recent research also highlights AI's potential to support mental health care, especially through AI-driven conversational agents that provide psychoeducational resources and mediate evidence-based therapeutic techniques (68, 69). Finally, participants also discussed reasons why AI remains underutilized in some fields. Insufficient data, data protection concerns, and technical challenges were identified as major barriers. These issues reflect findings in literature highlighting the need for robust datasets and regulatory frameworks to advance AI in healthcare (55, 70).

The findings of this study offer key insights into AI integration in medical care and highlight important implications for research and practice. They emphasize factors influencing physicians’ acceptance, such as efficiency gains, while underscoring the essential role of expert judgment and autonomous decision-making authority of physicians. We suggest that a key consideration for the integration of AI into medical practice should be to ensure that it complements, rather than replaces, physician decision-making, while preserving physician autonomy and medical judgment. Concerns about AI's impact on the physician–patient relationship suggest that implementation should prioritize human interaction and trust. Additionally, challenges related to standardization, and data protection require clear regulatory frameworks, such as the recently enacted AI Act, a European regulation aimed at ensuring legal and ethical standards for AI development and implementation enabling AI's social acceptance across diverse populations (23, 70). This initiative marks a step forward in building confidence among AI providers and users (71). Our study contributes to the broader discourse on AI prerequisites for implementation and acceptance in medical care by offering a cross-specialty perspective, filling a gap in previous studies that were limited to individual specialties and lacked a broader view (31, 34, 72). The study identifies avenues for future research, particularly on how AI can be integrated into specific medical domains, such as mental health and situations involving complex clinical decisions. These include, for example, end-of-life care, resource allocation, or treatment prioritization, where the unique challenges of patient sensitivity and moral dilemmas need to be addressed. Additionally, future studies could employ a quantitative methodology to determine the impact of influencing factors, such as specialization, on physicians’ attitudes and to explore the broader impact of AI integration in medical practice. As part of the broader PEAK study, patients also participated in focus groups exploring their perceptions of AI in medical care (73). Their views aligned in several respects with those of physicians, especially regarding the importance of empathy and human oversight. In light of recent advances in conversational diagnostic systems (74), future research should also investigate how patients experience empathy and communication in interactions with AI-based systems, and how these perceptions compare to those of physicians. Building on physicians’ concerns about preserving trust and human connection in the physician–patient relationship, future studies should also explore how AI might support shared decision-making by encouraging more active patient involvement in treatment discussions and supporting patient autonomy (75). This will be essential to understanding how AI-mediated shared decision-making influences adherence, satisfaction, and the evolving roles of both patients and physicians in medical care (66, 76). Given the rapid pace of AI developments in medicine, conducting repeated studies at regular intervals is recommended to capture evolving physician attitudes and implementation conditions. This is particularly relevant in light of expanding AI-related training opportunities, such as the German AI education platform KI Campus, which offers Continuing Medical Education (CME)-certified courses for physicians (77). Addressing these questions is essential to ensure the responsible and effective application of AI in medical care.

## Strengths and limitations

A strength of this study is its inclusion of both hospital physicians and GPs, providing insights from multiple medical specialties on the implementation of AI in medical care. By employing a qualitative approach, the study allowed for a more nuanced analysis of physicians’ opinions. However, it is important to note some limitations of this study. The gender distribution among participants, particularly the overrepresentation of male GPs, may have influenced the diversity of perspectives captured. This imbalance may stem from differing interest in AI-related topics or time constraints, as the two-hour focus group duration may have posed a greater barrier for some participants (78). Previous research suggests, however, that gender does not necessarily influence attitudes towards AI (79). This study did not include physicians working in secondary care settings, such as outpatient specialists, whose perspectives may differ and should be explored in future research. As most participating physicians had little to no experience with AI in their daily medical practice, the discussions were largely based on hypothetical scenarios, which should be considered when interpreting the findings. Another limitation is the potential for selection bias, as participation in the FGs was voluntary, which may have led to an overrepresentation of physicians with a particular interest in AI. Given that the FGs were conducted both before and after the public release of ChatGPT in November 2022, the accompanying media attention may have further increased general interest in AI. Notably, ChatGPT was mentioned a few times when participants were asked about their initial associations with AI, indicating a general awareness of its emergence. However, the fact that the majority of study participants demonstrated a high level of technical affinity suggests that this external influence, if any, was likely minimal. Despite these limitations, the study provides a valuable starting point for further research and highlights important gaps in the action needed for meaningful AI implementation in medical care.

## Conclusion

This qualitative study provides insights into how German physicians from various specialties perceive the use of AI in medical care, revealing both an openness toward its application and concerns about potential risks. These included organizational, regulatory, and ethical challenges, as well as the need for human oversight. Physicians emphasized interpersonal relationships as irreplaceable by AI, highlighting the importance of preserving human interaction when integrating AI into medical care. Addressing these concerns is key to successful implementation, with physicians’ attitudes central to shaping effective AI applications.

## Data Availability

The raw qualitative data supporting the conclusions of this article will be made available by the authors, in compliance with applicable ethical and privacy standards.
